# Is preoperative imaging of sentinel lymph node in breast cancer necessary? A retrospective case control study

**DOI:** 10.1016/j.breast.2024.103856

**Published:** 2024-12-11

**Authors:** Michael J. Reinhardt, Björn Ohmstede, Luz Angela Torres-de la Roche, Rudy Leon De Wilde

**Affiliations:** a Clinic for Nuclear Medicine, Pius Hospital, University Medicine Oldenburg, Carl von Ossietzky University Oldenburg, 26121, Germany; b Department of Anaesthesiology and Intensive Care Medicine, Pius Hospital, University Medicine Oldenburg, Carl von Ossietzky University Oldenburg, 26121, Germany; c University Hospital for Gynecology, Pius Hospital, University Medicine Oldenburg, Carl von Ossietzky University Oldenburg, 26121, Germany

**Keywords:** Sentinel lymph node, Breast cancer, Scintigraphy

## Abstract

**Objective:**

The necessity of preoperative lymphoscintigraphy before intraoperative sentinel lymph node (SLN) identification is still unclear. The aim of the present study was to evaluate the impact of SLN imaging on intraoperative SLN detection in breast cancer patients.

**Methods:**

Retrospective, comparative, single center study of patients with breast cancer stage pT1 and pT2 who underwent axillary staging. Group 1 included patients who underwent SLN extirpation without preoperative SLN imaging, and Group 2 included patients who underwent SLN imaging prior to surgery. Differences between groups were analyzed using T-test and chi-square test.

**Results:**

926 (mean age: 61.45 years) patients received subdermal injection of radiolabeled nanocolloids between tumor and axilla. SLN was identified intraoperatively in 473 of 498 patients (94.98 %) in group 1, and in 415 of 428 patients (96.96 %) in group 2 (p = 0.129). Lymphoscintigraphy detected SLN in 407 of 428 (95.09 %) patients in group 2. Due to the additional SLN imaging in group 2, the time between radiotracer injection and start of surgery was significantly prolonged (p < 0.001). A reduction of applied activity from a median of 18 MBq in group 1 to a median of 12 MBq in group 2 had no negative effect on the SLN identification.

**Conclusion:**

Subdermal injection of radiolabeled nanocolloids between tumor and axilla allowed high intraoperative detection of SLN. Preoperative SLN imaging had no significant impact on the intraoperative detection but is a time-consuming and resource-intensive procedure. Prospective studies might provide further evidence to omit preoperative SLN imaging in patients with T1-2 invasive breast cancer and clinically negative axilla.

## Highlights

• Removal of sentinel lymph nodes is a key concept in breast cancer.
• Preoperative scintigraphy is often performed prior to SLN extirpation.
• The contribution of imaging to intraoperative SLN identification is unclear.
• There was no benefit of preoperative scintigraphy for intraoperative SLN detection.

## 1 Introduction

Breast cancer (BC) is the most common malignant tumor in women worldwide, accounting for 23.8 % of all cancers in 2020 [1]. Patients with tumors of less than 5 cm benefit from less radical and therefore less compromising treatment with sentinel lymph node biopsy (SLNB), instead of level I/II axillary lymph node dissection (ALND), recommended for patients with T1-2 invasive BC and a clinically negative axilla [2].The success of SLNB is comparable in a variety of injection sites (peritumoral, intratumoral, intradermal, subdermal, subareolar) and injection volumes. Isotope mapping has been reported to be superior to that of blue dye [2]. SLNB can be performed with and without preoperative scintigraphic imaging. However, there is no consensus about the impact of preoperative lymphoscintigraphy. For example, Goyal et al., 2005 concluded that preoperative scintigraphy of sentinel lymph node (SLN) significantly improves intraoperative recovery [3]. In contrast, Sun et al., 2010 did not describe a higher intraoperative recovery of the SLN with preoperative SLN scintigraphy [4]. Consequently, national and international guidelines and recommendations show no uniformity regarding preoperative SLN imaging.

In 2013 the European and American Societies of Nuclear Medicine (EANM, SNMMI) issued a joint recommendation that SLNB may include preoperative scintigraphic imaging [5] and in 2014, the German Society of Nuclear Medicine (DGN) expected preoperative SLN detection in 95 % of patients [6]. The main considerations were quality control and quality assurance [5,6]. Therefore, we implemented SLN scintigraphy 2014 to SLNB at our German Cancer Society (DKG) certified BC Centre. However, little has been done since then to evaluate the role of preoperative SLN imaging on the intraoperative detection rate of SLN.

In 2021, the DKG gave a recommendation for SLNB in the context of axillary staging with recommendation grade A and level of evidence 1a [7], but a recommendation for preoperative SLN scintigraphy was again not issued.

Therefore, the aim of the present study was to evaluate the impact of preoperative SLN scintigraphy on intraoperative SLN detection rate.

## 2 Methods

### 2.1 Patients

A retrospective, single center, case-control study. Approval from the Medical Ethics Committee of the Carl von Ossietzky University of Oldenburg was obtained prior to study initiation (file number 2017-053).

Between January 2012 and December 2016, 1315 patients with biopsy proven BC without clinical evidence of axillary lymph-node metastases received SLNB at our DKG certified BC center. 389 patients with either DCIS, neoadjuvant radiotherapy, recurrent disease, or higher postoperative tumor stages than T2 as well as multicentric and inflammatory tumors, were excluded. 926 patients with T1 and T2 stages were eligible for further evaluation. Preoperative SLN imaging was added to SLNB at 01.09.2014. Two consecutive groups were formed: group 1 included 498 patients from 01.01.2012 until 30.08.2014, who underwent SLNB without preoperative SLN imaging after radiotracer injection and group 2 included 428 patients from 01.09.2014 until 31.12.2016, who underwent SLNB with additional preoperative SLN imaging after radiotracer injection at the same day (one-day protocol).

### 2.2 Tracer injection, imaging and surgery

A single injection with a standard activity of median 12 or 18 ^99m^Tc-labelled human albumin nanocolloid particles with a particle size of approximately 20–100 nm (NanoHSA-ROTOP/NANOTOP®) were applied subdermally within 2 cm distance to the tumor. The radiotracer was administered by four breast surgeons with an individual experience in SLNB-Procedure of more than 5 years each prior to the present evaluation. Approximately 250 SLNB were performed each year at our institution. Group 1 patients were subsequently prepared for surgery. In group 2, static images were then taken 30 min after injection in anterior and lateral view according to our institutional protocol. Siemens™ E.CAM® single headed gamma camera equipped with low energy high resolution collimator was used. If the SLN was not visible after 30 min, later images were performed after active and/or passive warming of the injection site. During axillary sentinel lymph node dissection (SLND), the skin was incised at the anterior axillary line or a transmammary entrance was used. The axillary lymphatic fatty tissue was dissected and the axillary SLN with the highest count rate was detected using a hand-held gamma probe (HiSens® probe, Crystal Photonics GmbH, Berlin) and removed. After that the axilla was searched for residual lymph nodes with a signal higher than that of surrounding tissue. These lymph nodes were also removed. We did not combine radiotracer and blue dye injection. All dissected lymph nodes were sent to pathology analysis afterwards.

### 2.3 Statistical analysis

All data were pseudonymized and transferred to an Excel spreadsheet (Microsoft® Excel for Mac) and analyzed in a database structure for biostatistical analysis using IBM® SPSS® Statistics versions 24–27 for Mac. Descriptive statistics with calculation of frequencies for patient characteristics and outcomes were performed, including age at the time of surgery, body weight, tumor localization, tumor size and stage, number of SLN, applied ^99m^Tc activity, time between tracer injection and start of surgery as well as the frequency of preoperative and intraoperative SLN detection. For comparisons between groups, the T-test with a 2-sided p-value was used for the comparison of data with normal distribution or data that are not normally distributed for groups greater than 100. The Pearson's chi-square test was used to determine the correlation between two categorical variables. The global significance level chosen was p < 0.05.

## 3 Results

### 3.1 Patient and tumor characteristics

Student's T-Test comparing the characteristics of the patients and tumors showed no significant difference between groups regarding age, body weight and tumor size (Table 1). Pearson's Chi-Square test showed no significant difference between groups regarding tumor stage T1 and T2 (p = 0.493) but for tumor localization (p = 0.035), because group 1 comprised significantly more left sided tumors than group 2 (56.2 % versus 49.3 %). There was no change in the performing physicians, the injection technique, the imaging or the surgical protocol during the entire study.

**Table 1:** Patient's and tumor characteristics.

	Total (n = 926)	Group 1 (n = 498)	Group 2 (n = 428)	p-value (test)
**Age [years]**				
Mean (SD)	61.45 (12,156)	60.91 (12.247)	62.08 (12.033)	0.145 (T-Test)
**Tumor stage**				
pT1	640 (69.1 %)	349 (70.08 %)	291 (67.99 %)	0.493 (Chi^2^-Test)
pT2	286 (30.9 %)	149 (29.92 %)	137 (32.01 %)	
**Body weight [kg]**				
Mean (IQR)		74.09 (63.00–83.00)	75.14 (63.00–83.00)	0.345 (T-Test)
**Tumor size [mm]**				
Mean (IQR)		16 [[11], [12], [13], [14], [15], [16], [17], [18], [19], [20], [21], [22], [23]]	17 [[12], [13], [14], [15], [16], [17], [18], [19], [20], [21], [22], [23], [24], [25]]	0.76 (T-Test)
**Tumor localization**				
Left breast	491 (53.02 %)	280 (56.2 %)	211 (49.3 %)	0.035 (Chi^2^-Test)
Right breast	435 (46.98 %)	218 (43.8 %)	217 (50.7 %)	

SD: standard deviation, IQR: interquartile range.

### 3.2 SLN detection

**Fig. 1:**
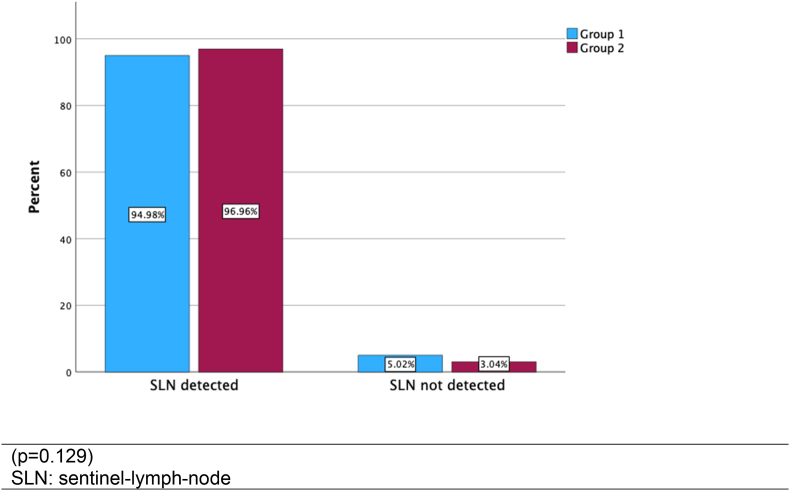
Lymph node detection rate in dependence on pre-operative imaging.

Axillary SLN was identified and retrieved intraoperatively in 473 of 498 patients (94.98 %) in group 1 and in 415 of 428 patients (96.96 %) patients in group 2 (Fig. 1). The difference in the Pearson's Chi-Square-Test was not significant (p = 0.129). In group 1, a single SLN was detected in 62.45 %, two SLN in 29.12 % and three SLN in 3.41 %. In group 2, a single SLN was detected in 81.07 %, two SLN in 14.25 % and three SLN in 1.64 %. Preoperative lymphoscintigraphy detected axillary SLN in 407 of 428 patients (95.09 %) in group 2. Extra axillary lymph nodes were not considered for evaluation.

SLN was further detected intraoperatively in 623 of 640 patients with pT1 stage (97.28 %) and in 265 of 286 patients with pT2 stage (92.08 %). The difference in Pearson's Chi-Square-Test was significant (p < 0.001). There was no difference between intraoperative SLN detection in pT1 and pT2 stage when analyzed separately for group 1 and 2 (p = 0.419).

In group 2, the axillary SLN were not found intraoperatively in 9 cases (2.10 %) despite a focal tracer accumulation was seen on scintigraphy (false positive). 407 cases (95.09 %) were correctly identified on scintigraphy and the SLN removed (true positive). Differences regarding post-surgical complications in group 1 and 2 were not reported during the short hospitalization of patients as the surgical procedure was identical.

### 3.3 Applied activity

**Fig. 2:**
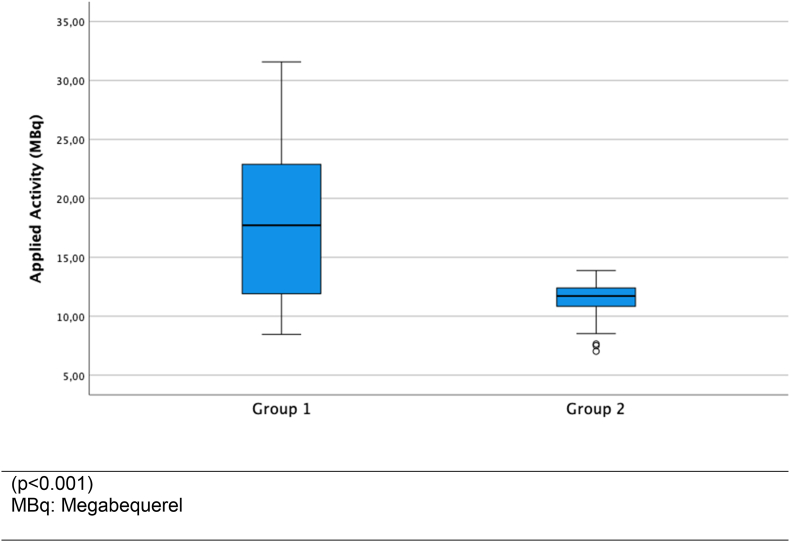
Administered net activity for SLN identification.

The net activity applied was calculated for 919 of 926 patients because in 7 patients the remaining activity in the syringe after injection was not documented (5 patients of group 1 and 2 patients of group 2). The applied activity in group 1 was 17.83 MBq ± 5.65 MBq, median 17.72 MBq, interquartile range 11.90–22.90 MBq and in group 2 it was 11.57 MBq ± 1.13 MBq, median 11.72 MBq, interquartile range 10.83–12.72 MBq (Fig. 2). The difference in the Mann-Whitney *U* test was significant (p < 0.001), showing a more heterogeneous net activity distribution in group 1. Therefore, we analyzed whether SLN detection depended on the activity applied. The mean activity in 882 patients with SLN detection was 14.91 MBq ± 5.23 MBq, median 12.25 MBq, interquartile range 10.97–21.58 MBq and in 37 patients without SLN detection it was 15.25 MBq ± 5.52 MBq, Median 12.38 MBq, interquartile range 11.31–18.11 MBq. The difference in the Mann-Whitney *U* test was not significant (p = 0.933).

### 3.4 Time between injection and start of surgery

**Fig. 3:**
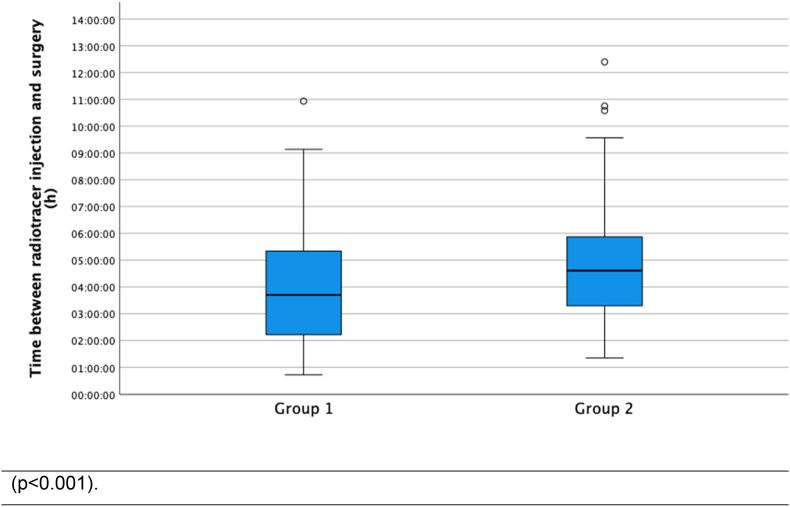
Time between radiotracer injection and start of surgery in dependence on pre-operative sentinel lymph node imaging.

The time between radiotracer injection and start of SLNB was in mean 3:52±2:04 h for group 1 and 4:44±1:44 h for group 2. The difference in the T-test was significant (p < 0.001) (Fig. 3).

## 4 Discussion

The usefulness of preoperative SLN scintigraphy in BC is a topic of controversy in the literature. In this analysis we systematically compared the intraoperative SLN detection rate with and without preoperative SLN imaging. During the 5-year study period, almost all potential factors influencing intraoperative SLN identification [2, 5, 8], such as personnel, technical and procedural standards, remained constant. This is the most significant strength of this study. Regarding the intraoperative detection rate, the difference observed between group 1 (94,98 %) and group 2 (96,96 %) was not statistically significant (p = 0.129). The only significant differences between both groups were a higher activity administered in group 1 and a longer time interval between tracer injection and the start of surgery in group 2. The frequencies achieved were similar to other studies reporting detection rates of 72 % [3], 81.7 % [8], 86.5 % [9] and 91.4 % [10], and consistent with the expected rates of the DGN [6].

Some previous studies have reported that preoperative SLN imaging improves the rate of intraoperative axillary SLN localization. Goyal et al. used a combination of radioactive tracer and blue dye in 823 patients and detected SLN on preoperative lymphoscintigraphy in 593 patients (72 %), most of them in the axilla (96 %) [3]. 98 % of SLN visualized on lymphoscintigraphy were detected intraoperatively. In 90 % of the remaining 230 patients without preoperative SLN detection, the SLN was detected intraoperatively. However, the authors did not have a control group without preoperative imaging because all patients received lymphoscintigraphy.

In 2006, Marchal et al. described a highly significant influence of preoperative SLN scintigraphy on intraoperative axillary SLN identification in 201 patients using a combination of radioactive tracer and blue dye (p < 0.001) [11]. All patients received ALND after SLN biopsy. Again, the authors did not have a control group without preoperative imaging.

In their retrospective study from 2007, Wang et al. identified SLN in 86.5 % of 636 patients with preoperative SLN imaging and detected 625 SLN (98.3 %) intraoperatively. They concluded, that preoperative lymphoscintigraphy predicted the successful SLN identification but was less informative for the location of SLN during operation and therefore was not necessary for SLNB [9].

Several other studies reported that preoperative lymphoscintigraphy did not improve the ability to identify axillary SLN during surgery. A study by McMasters et al., in 2000 found no positive effect of lymphoscintigraphy on intraoperative axillary SLN detection in 588 patients, who underwent SLNB after radiotracer injection followed by level I/II axillary dissection. SLN was identified during surgery in 310 of 348 patients (89.1 %) with and in 221 of 240 patients (92.1 %) without preoperative lymphoscintigraphy [12].

In their 2000 review, Alazraki et al. concluded that preoperative SLN scintigraphy was inferior to the use of the hand-held gamma probe alone in terms of intraoperative SLN identification [13].

In 2010, Sun et al. described in their prospective randomized study of 565 patients that preoperative SLN imaging did not improve the intraoperative detection rate. In this study, two groups with and without preoperative SLN scintigraphy were compared. In the group with imaging, the SLN was identified preoperatively in 238 patients (82.1 %), with an intraoperative detection rate of 99.3 %. In the group without imaging, the SLN was found intraoperatively in 99.6 % of cases. The difference was not significant [4].

In their 2019 prospective multicentric study of 1,158 patients, Kuemmel et al. reported a mean number of histologically detected axillary SLN of 2.21 with lymphoscintigraphy and 2.26 without lymphoscintigraphy. The authors concluded, that SLNB is equally effective irrespective of the surgeon's knowledge of preoperative lymphoscintigraphy results and that SLNB without preoperative SLN scintigraphy will speed up the preoperative workflow and reduce cost [14].

Several papers addressed the usefulness of combined techniques [15,16]. Radiotracer injection can be used in combination with the injection of a color dye or fluorescent agent, which is supposed to be the gold-standard [17]. However, the results depended on training and experience of the surgeons and skin tattoos and adverse reactions to the dye followed often. Indocyanine green (ICG) is a promising new agent that has been developed in recent years. A meta-analysis from 2021 considered the use of ICG to be superior to that of blue dye and equivalent to the use of radiotracers [18].

Radiotracer application can be done at the same day as SLNB or the day before. A two-day protocol allows for more flexible scheduling of patients for surgery and a longer time for the radiotracer to accumulate in the SLN. A one-day protocol as used in our institution has the advantages of a lower radiation burden to the patient and a shorter hospital stay. However, there is no significant difference between the protocols concerning the detection rate of SLN using a probe or scintigraphy [19].

Complications after SLNB are less frequent compared to ALND. However, no study compares the change in morbidity achieved by lymphoscintigraphy vs. the use of a hand-held gamma probe alone [20]. This review from 2005 emphasized a reduced SLNB-associated morbidity when preoperative imaging is performed [20]. However, studies performing ALND as well as SLNB were included and not differentiated concerning their impact on postsurgical complications. Thus, significant less pain and paresthesia reported in the imaging studies might be a result of less extensive surgical procedures, i.e. less ALND. Unfortunately, we cannot provide data about complications after SLNB because we could not differentiate them reliably from those derived from breast surgery, which was usually performed the same day.

Regarding the injection technique, the debate about the best injection site is still ongoing and some authors see advantages in subdermal application. They pointed to the simplier technique, a shorter radiocolloid uptake time and higher radiocolloid uptake in the SLN [21]. The FRANSENODE study found advantages for the periareolar injection technique with a higher detection rate in preoperative SLN scintigraphy and higher intraoperative identification of at least one SLN [8].The intraoperative detection rate of periareolar injection in the FRANSENODE study was only slightly higher compared to the results obtained in the present study (98 % vs 95 %), which used a subdermal injection between the tumor and the axilla [8].

In the present analysis a significant difference was observed in the applied activity of ^99m^Tc-nanocolloids with a median of 17,7 MBq in group 1 and a median of 11,7 MBq in group 2. This difference was explained by the reduction of applied activity indicated by the "Guideline on radiation protection in medicine" (dated 25/05/2011), as amended on 11/07/2014, which required not more than 10 MBq of ^99m^Tc-labelled nanocolloids at the time of surgery [21]. Consequently, patients in group 2 received a lower dose. However, there was no significant difference in intraoperative SLN identification based on the activities applied between the two groups.

The EANM/SNMMI guideline state that preoperative sentinel node mapping improves accuracy and reduces the morbidity relative to the use of the hand-held gamma probe alone [5]. However, this statement refers to a single feasibility study from 1997 including 37 patients, who received SLN imaging 2 and 18 h after radiotracer injection and were referred to ALND the day after radiotracer injection [22]. 89 % of SLN were seen on the images after 2 h and 92 % of SLN were seen on the images after 18 h. This study is compromised by the fact, that the authors did not have a control group without imaging and that they did not report about the morbidity due to the ALND procedure, which is usually higher than that of the SLNB procedure performed nowadays.

Even in our study, we observed a slight but not significant higher SLN detection rate of 96.96 % in group 2 with a longer time of 4:44±1:44 h between radiotracer injection and surgery compared to 94.98 % in group 1 (p < 0.129) with a significantly shorter time of 3:52±2:04 h between radiotracer injection and surgery (p < 0.001).

In their 2013 guideline, the EANM and SNMMI stated that consensus exists on some broad aspects of SLN protocols in BC, which may include preoperative scintigraphic imaging, but not on all details [5]. A detection rate for SLN identification is not given. The DKG claims an intraoperative SLN detection rate of at least 90 % as part of the accreditation as a BC Center, but preoperative SLN scintigraphy is optional [23]. Using standard protocols, SLN should be identified in 95 % of eligible patients according to the American Society of Breast Surgeons [2]. The European Society for Medical Oncology (ESMO) stated in 2015, that high intraoperative SLN identification rates of 97 % are achievable using a combination of methods. Preoperative lymphoscintigrams were not needed [24]. Further guidelines notice that the added information of preoperative SLN scintigraphy is limited [25] or can be done at the discretion of the nuclear medicine physician and surgeon [2] or do not even mention the possibility of preoperative SLN imaging at all [7,26,27]. The results obtained in the present analysis indicate that preoperative imaging does not provide significant benefits for identification of axillary SLN during BC staging surgery.

The EANM/SNMMI guideline sees the benefits of preoperative lymphoscintigraphy in terms of quality control, as an efficient means of determining if there is uptake of activity in any node and to improve the likelihood of identifying and locating all relevant nodes [5]. Unfortunately, the EANM/SNMMI guideline and the German S1 guideline have not been updated since their publication more than 10 years ago. Evidence-based literature of later years is therefore not considered. Many papers cited in these guidelines are just proof-of-concept studies. Thus, these guidelines can be characterized rather as an expert consensus than as an evidence-based guideline.

Under the terms of quality assurance, the DGN guideline adds the expectation of successful preoperative SLN detection in 95 % of cases using an optimized technique [6]. Quality control and quality assurance are crucial components of quality management (QM). A central principle of QM is process orientation [28], placing a priority on how measures are done. Process-oriented QM can look back on a long-term development that, in the sense of a continuous improvement, a process can never be completely finished, but always has to be re-evaluated and, eventually, further developed [29]. Even guidelines, procedural instructions and professional recommendations should be understood as a process, which aims at continuous improvement according to the PDCA cycle (Plan-Do-Check-Act) [28]. The expectation of 95 % preoperative SLN detection has never been validated nor revised [6]. In terms of a PDCA cycle this postulation is just in its second phase.

The goal of SLNB is to accurately stage the axilla. Our data show that preoperative SLN scintigraphy has no impact on this goal in the hands of an experienced team. Preoperative lymphoscintigraphy could therefore, eventually, be omitted. The QM process should now enter its third phase of PDCA cycle and check if the expectation of 95 % preoperative SLN identification should continue at all. After that, the fourth phase has to follow and the procedure should be changed accordingly.

The study presented here is not a prospective randomized multicenter study. This is the most significant limitation of this study. Our study design is a monocentric retrospective case-control study which can suggest causal relationships but cannot prove them.

## 5 Conclusion

The use of preoperative radiotracer lymphoscintigraphic mapping has been strongly advocated due to the potential for enhanced accuracy and reduced morbidity when compared to the use of a hand-held gamma probe alone. However, the presented data suggest that preoperative axillary SLN scintigraphy does not improve intraoperative axillary SLN detection during BC staging surgery in the hands of an experienced team, but is a time-consuming and resource-intensive procedure. Subdermal injection of 10–13 MBq ^99m^Tc-labelled nanocolloids between tumor and axilla allows a high intraoperative SLN detection at the time of surgery. Prospective studies might provide further evidence to support the option of omitting preoperative SLN scintigraphy in patients with T1 and T2 invasive BC with a clinically negative axilla.

## CRediT authorship contribution statement

**Michael J. Reinhardt:** Writing – review & editing, Writing – original draft, Supervision, Methodology, Investigation, Formal analysis, Conceptualization. **Björn Ohmstede:** Writing – review & editing, Writing – original draft, Formal analysis, Data curation. **Luz Angela Torres-de la Roche:** Writing – review & editing, Writing – original draft, Validation. **Rudy Leon De Wilde:** Writing – review & editing, Project administration, Conceptualization.

## Funding

No funds were received for this analysis.

## Declaration of competing interest

The authors declare that they have no competing financial interests or personal relationships that could influence the work reported in this paper.

## References

[1] WHO, International Agency for Research on Cancer (2022). Incidence of cancer, Females, in 2022. https://gco.iarc.fr/today/en/dataviz/pie?mode=cancer&group_populations=1&sexes=2&cancers=20.

[2] The American Society of Breast Surgeons (2014). Performance and practice guidelines for sentinel lymph node biopsy in breast cancer patients: the American society of breast surgeons. https://www.breastsurgeons.org/docs/statements/Performance-and-Practice-Guidelines-for-Sentinel-Lymph-Node-Biopsy-in-Breast-Cancer-Patients.pdf.

[3] Goyal A., Newcombe R.G., Mansel R.E., Chetty U., Ell P., Fallowfield L. (2005). Role of routine preoperative lymphoscintigraphy in sentinel node biopsy for breast cancer. Eur J Cancer.

[4] Sun X., Liu J.J., Wang Y.S., Wang L., Yang G.R., Zhou Z.B. (2010). Roles of preoperative lymphoscintigraphy for sentinel lymph node biopsy in breast cancer patients. Jpn J Clin Oncol.

[5] Giammarile F., Alazraki N., Aarsvold J.N., Audisio R.A., Glass E., Grant S.F. (2013). The EANM and SNMMI practice guideline for lymphoscintigraphy and sentinel node localization in breast cancer. Eur J Nucl Med Mol Imag.

[6] Schmidt M., Bares R., Brenner W., Buck A. (2014). Verfahrensanweisung für die technische Durchführung der nuklearmedizinischen Wächter-Lymphknoten-Diagnostik. https://www.nuklearmedizin.de/leistungen/leitlinien/docs/031-033l_S1_Waechter_Lymphknoten_Diagnostik_2014-10.pdf.

[7] Krebsgesellschaft Deutsche, Krebshilfe Deutsche, AWMF (2020). Leitlinienprogramm Onkologie: S3-Leitlinie Früherkennung, Diagnose, Therapie und Nachsorge des Mammakarzinoms. http://www.leitlinienprogramm-onkologie.de/leitlinien/mammakarzinom/.

[8] Rodier J.F., Velten M., Wilt M., Martel P., Ferron G., Vaini-Elies V. (2007). Prospective multicentric randomized study comparing periareolar and peritumoral injection of radiotracer and blue dye for the detection of sentinel lymph node in breast sparing procedures: FRANSENODE trial. J Clin Oncol.

[9] Wang L., Yu J-m, Wang Y-s, Zuo W-s, Gao Y., Fan J. (2007). Preoperative lymphoscintigraphy predicts the successful identification but is not necessary in sentinel lymph nodes biopsy in breast cancer. Ann Surg Oncol.

[10] Namwongprom S., Boonyaprapa S., Ekmahachai M., Vilasdechanon N., Somwangprasert A., Sumitsawan S. (2005). Breast lymphoscintigraphy for sentinel node identification in breast cancers with clinically-negative axillary nodes. Singapore Med J.

[11] Marchal F., Rauch P., Morel O., Mayer J.C., Olivier P., Leroux A. (2006). Results of preoperative lymphoscintigraphy for breast cancer are predictive of identification of axillary sentinel lymph nodes. World J Surg.

[12] McMasters K.M., Wong S.L., Tuttle T.M., Carlson D.J., Brown C.M., Dirk Noyes R. (2000). Preoperative lymphoscintigraphy for breast cancer does not improve the ability to identify axillary sentinel lymph nodes. Ann Surg.

[13] Alazraki N.P., Styblo T., Grant S.F., Cohen C., Larsen T., Aarsvold J.N. (2000). Sentinel node staging of early breast cancer using lymphoscintigraphy and the intraoperative gamma-detecting probe: sentinel Node Localization. Semin Nucl Med.

[14] Kuemmel S., Holtschmidt J., Gerber B., Von der Assen A., Heil J., Thill M. (2019). Prospective, multicenter, randomized phase III trial evaluating the impact of lymphoscintigraphy as part of sentinel node biopsy in early breast cancer: SenSzi (GBG80) trial. J Clin Oncol.

[15] Motomura K., Inaji H., Komoike Y., Hasegawa Y., Kasugai T., Noguchi S. (2001). Combination technique is superior to dye alone in identification of the sentinel node in breast cancer patients. J Surg Oncol.

[16] McMasters K.M., Tuttle T.M., Carlson D.J., Brown C.M., Noyes R.D., Glaser R.L. (2000). Sentinel lymph node biopsy for breast cancer: a suitable alternative to routine axillary dissection in multi-institutional practice when optimal technique is used. J Clin Oncol.

[17] Ferrucci M., Franceschini G., Douek M. (2018). New techniques for sentinel node biopsy in breast cancer. Transl Cancer Res.

[18] Kedrzycki M.S., Leiloglou M., Ashrafian H., Jiwa N., Thiruchelvam P.T.R., Elson D.S. (2021). Meta-analysis comparing fluorescence imaging with radioisotope and blue dye-guided sentinel node identification for breast cancer surgery. Ann Surg Oncol.

[19] van Esser S., Hobbelink M., Van Isselt J.W., Mali W.P., Borel Rinkes I.H., van Hillegersberg R. (2009). Comparison of a 1-day and a 2-day protocol for lymphatic mapping and sentinel lymph node biopsy in patients with nonpalpable breast cancer. Eur J Nucl Med Mol Imag.

[20] Krynyckyi B.R., Shafir M.K., Kim S.C., Kim D.W., Travis A., Moadel R.M. (2005). Lymphoscintigraphy and triangulated body marking for morbidity reduction during sentinel node biopsy in breast cancer. Int Semin Surg Oncol.

[21] Povoski S.P., Olsen J.O., Young D.C., Clarke J., Burak W.E., Walker M.J. (2006). Prospective randomized clinical trial comparing intradermal, intraparenchymal, and subareolar injection routes for sentinel lymph node mapping and biopsy in breast cancer. Ann Surg Oncol.

[22] Pijpers R., Meijer S., Hoekstra O.S., Collet G.J., Comans E.F.I., Boom R.P.A. (1997). Impact of lymphoscintigraphy on sentinel node identification with Technetium-99m-Colloidal albumin in breast cancer. J Nucl Med.

[23] Deutsche Krebsgesellschaft (2023). Erhebungsbogen Brustkrebszentren. https://www.krebsgesellschaft.de/zertdokumente.html.

[24] Senkus E., Kyriakides S., Ohno S., Penault-Llorca F., Poortmans P., Rutgers E. (2015). Primary breast cancer: ESMO Clinical Practice Guidelines for diagnosis, treatment and follow-up. Ann Oncol.

[25] Janni W. (2020). Munich..

[26] Lyman G.H., Somerfield M.R., Bosserman L.D., Perkins C.L., Weaver D.L., Giuliano A.E. (2017). Sentinel lymph node biopsy for patients with early-stage breast cancer: American society of clinical Oncology clinical practice guideline update. J Clin Oncol.

[27] Gradishar W.J., Anderson B.O., Balassanian R., Blair S.L., Burstein H.J., Cyr A. (2017). NCCN guidelines insights: breast cancer, version 1.2017. Journal of the National Comprehensive Cancer Network J Natl Compr Canc Netw.

[28] Nix O., Schlegel W., Karger C.P., Jäkel O. (2018). Medizinische Physik: Grundlagen – Bildgebung – Therapie – Technik.

[29] Kohlbacher M. (2010). The effects of process orientation: a literature review. Bus Process Manag J.

